# Uncovering a Cryptic Site of Malaria Pathogenesis: Models to Study Interactions Between *Plasmodium* and the Bone Marrow

**DOI:** 10.3389/fcimb.2022.917267

**Published:** 2022-06-02

**Authors:** Tamar P. Feldman, Elizabeth S. Egan

**Affiliations:** ^1^ Department of Pediatrics, Stanford University School of Medicine, Stanford, CA, United States; ^2^ Department of Microbiology and Immunology, Stanford University School of Medicine, Stanford, CA, United States

**Keywords:** *Plasmodium*, bone marrow, hematopoietic stem cells, erythropoiesis, malaria anemia, *ex-vivo* erythropoiesis model

## Abstract

The bone marrow is a critical site of host-pathogen interactions in malaria infection. The discovery of *Plasmodium* asexual and transmission stages in the bone marrow has renewed interest in the tissue as a niche for cellular development of both host and parasite. Despite its importance, bone marrow in malaria infection remains largely unexplored due to the challenge of modeling the complex hematopoietic environment *in vitro*. Advancements in modeling human erythropoiesis *ex-vivo* from primary human hematopoietic stem and progenitor cells provide a foothold to study the host-parasite interactions occurring in this understudied site of malaria pathogenesis. This review focuses on current *in vitro* methods to recapitulate and assess bone marrow erythropoiesis and their potential applications in the malaria field. We summarize recent studies that leveraged *ex-vivo* erythropoiesis to shed light on gametocyte development in nucleated erythroid stem cells and begin to characterize host cell responses to *Plasmodium* infection in the hematopoietic niche. Such models hold potential to elucidate mechanisms of disordered erythropoiesis, an underlying contributor to malaria anemia, as well as understand the biological determinants of parasite sexual conversion. This review compares the advantages and limitations of the *ex-vivo* erythropoiesis approach with those of *in vivo* human and animal studies of the hematopoietic niche in malaria infection. We highlight the need for studies that apply single cell analyses to this complex system and incorporate physical and cellular components of the bone marrow that may influence erythropoiesis and parasite development.

## Introduction

Malaria caused by *Plasmodium* spp. parasites remains a major global public health problem, responsible for an estimated 241 million cases and ~627,000 deaths per year (WHO, 2021). Of the five species that infect humans, *Plasmodium vivax* has the widest geographic spread whereas *Plasmodium falciparum* is the predominant cause of mortality. More than 90% of global malaria deaths are concentrated in endemic regions of western and sub-Saharan Africa. Although increased investments in effective anti-malarial treatment and disease prevention have led to marked improvements in malaria control over the past decade, the emergence of drug-resistant parasites and mosquitoes present continuing challenges.

*Plasmodia* are obligate, intracellular eukaryotic parasites transmitted by anopheline mosquitos and are characterized by a complex lifecycle in the mosquito and vertebrate host. Upon injection from the mosquito, parasites traffic to the liver where they undergo a massive, but clinically silent replication before entering the bloodstream. Symptoms of malaria occur exclusively during the blood stage of infection, when parasites invade and replicate exponentially in human erythrocytes. A small proportion of parasites commit to sexual reproduction, becoming male or female gametocytes. Circulating gametocytes can be transmitted to the mosquito vector as it takes a blood meal. Outside of circulation, *P. falciparum* and *P. vivax* can also be found in deep tissues, including the spleen and bone marrow.

The bone marrow is an important site of cellular development for both the parasite and its host. Post-natal hematopoiesis in humans occurs in the bone marrow where multipotent progenitors give rise to blood cell lineages, including red blood cells (RBCs) by a process termed erythropoiesis. Numerous case reports have demonstrated the presence of *Plasmodium* parasites, hemozoin, and abnormal erythroblast morphology in bone marrow biopsies of malaria patients (Abdalla et al., 1980; Dormer et al., 1983; Wickramasinghe et al., 1987). More recently, two quantitative studies using patient samples revealed that the bone marrow is a site of enrichment for sexual-stage gametocytes (Aguilar et al., 2014a; Joice et al., 2014), one of which also noted an association between severe anemia, dyserythropoiesis, and gametocyte load (Aguilar et al., 2014a). Together, these observations point to the bone marrow as an under-recognized site of malarial pathogenesis.

Although the observation of *Plasmodium* in human bone marrow dates back over 130 years (Marchiafava and Bigmani, 1894), our understanding of the impact of parasite infection on the hematopoietic niche is limited. The invasive procedure to obtain bone marrow aspirates, which is generally not clinically indicated for disease management, limits direct investigation in patient samples. Therefore, innovation in cell culture and animal models of erythropoiesis is crucial for understanding, and eventually disrupting, host-parasite interactions in the bone marrow that contribute to malaria pathogenesis. Here, we summarize available models for the study of host-parasite interactions in the hematopoietic niche, highlighting recent work that has leveraged advancements in *ex vivo* erythropoiesis of primary hematopoietic stem and progenitor cells (HSPCs) to generate and test hypotheses about the host response to parasite infection and parasite development in the bone marrow. Finally, we discuss avenues to improve existing models and the value of applying novel, single-cell technologies to the study of a complex tissue. Obtaining both cell-intrinsic and population-level views of how malaria parasites exploit and modulate the human bone marrow and specifically the erythroid lineage will lay the foundation for novel therapies for malaria.

## Overview of Human Erythropoiesis

**Figure 1:**
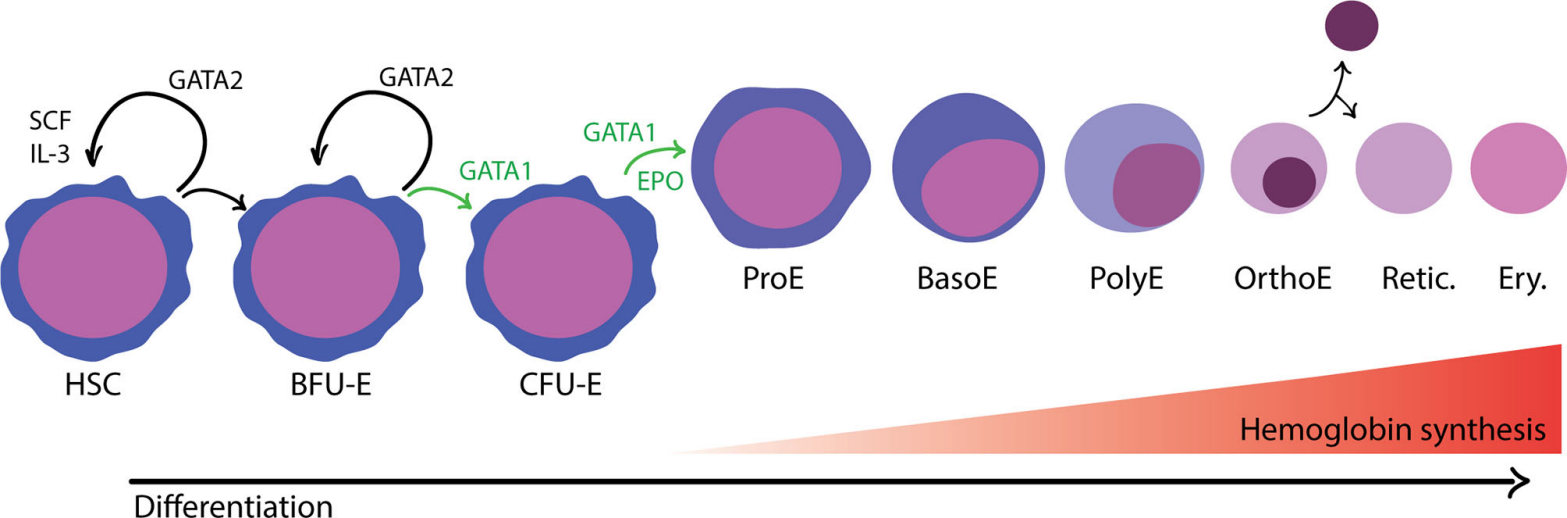
Overview of adult, definitive erythropoiesis in the bone marrow. The bone marrow is populated with hematopoietic stem cells with the capacity for long-term self-renewal and the potential to differentiate down any of the blood cell lineages. The balance between self-renewal and lineage commitment is informed by physical and chemical signals present in the surrounding microenvironment and coordinated by master transcription factors. Erythroid commitment is largely controlled by the activity of GATA1, which promotes differentiation and inhibits opposing factors for self-renewal, such as GATA2. In the first phase of development, progenitor cells become gradually more erythroid committed in response to environmental cues that promote proliferation and differentiation. Burst forming unit-erythroid (BFU-E) are considered the first irreversibly committed erythroid cells. In the subsequent stage, colony forming unit-erythroid (CFU-E) cells are highly proliferative and begin to activate erythroid transcriptional programs regulated by GATA1, in response to erythropoietin (EPO). In the terminal differentiation phase, erythroblasts develop in a specialized compartments called erythroblastic islands. Erythroblasts are found in close association with a central macrophage that provides signals to support proliferation and differentiation as well as the iron needed for hemoglobin synthesis. As erythroblasts differentiate, they decrease in size, undergo nuclear condensation, and turn off gene expression with the exception of a subset of erythroid-specific genes, most notably hemoglobin. Distinct morphological stages are visible by hematological staining: proerythroblast (ProE), basophilic erythroblast (BasE), polychromatic erythroblast (PolyE), and orthochromatic erythroblast (OrthoE). In the final stage of development, the nucleus and organelles are extruded. The enucleated reticulocyte (Retic.) undergoes further development as it enters circulation, eventually becoming a mature erythrocyte (Ery.).

In adult humans, erythropoiesis occurs in the extravascular compartment of the bone marrow and proceeds in two stages (Zivot et al., 2018) (**Figure 1**). First, multipotent hematopoietic stem and progenitor cells (HSPCs) lose their capacity to self-renew and become increasingly restricted in their lineage potential. The first erythroid committed progenitors, the burst forming unit-erythroid (BFU-E), give rise to colony forming unit-erythroid (CFU-E) (Hattangadi et al., 2011). The proliferation and survival of BFU-E, CFU-E, and proerythroblasts are promoted by stem cell factor (SCF), which binds to the c-KIT receptor (Sui et al., 2000). Erythropoietin (EPO), a hormone produced in the kidney, is an important regulator of erythropoiesis that balances the survival, proliferation, and differentiation of CFU-E. Binding of EPO to its receptor, EPOR, activates JAK2, which subsequently induces the activation of several cellular pathways including STAT5, RAS/MAP kinase and PI3K/AKT, thus promoting an erythroid lineage-restricted transcriptional program (Hattangadi et al., 2011; Caulier and Sankaran, 2022).

Terminal differentiation of erythroid cells proceeds through a limited number of cell divisions during which there are dramatic changes to cellular morphology, membrane properties, and gene expression (Zivot et al., 2018). As erythroblasts mature, cell size decreases, the nucleus condenses, and gene expression is gradually turned off, except for globin genes and other erythroid-specific factors. Finally, the orthochromatic erythroblast expels its nucleus and other organelles to become an enucleated reticulocyte. The reticulocyte undergoes further maturation and is released from the bone marrow into circulation, where it quickly matures into an erythrocyte (red blood cell; RBC).

## *Ex-vivo* Models of Human Erythropoiesis

The concept of generating human erythrocytes from hematopoietic stem cells by *ex-vivo* culture has long been a focus of the transfusion medicine field, with the goal of achieving a stable supply of mature red blood cells to meet worldwide clinical need (Anstee et al., 2012). To meaningfully recapitulate erythropoiesis, such an *in vitro* culture system needs to satisfy three criteria: large-scale proliferation of stem/progenitor cells, stable transition from multi-potent progenitors to commitment to the erythroid lineage, and successful terminal differentiation down the erythroid lineage to functional enucleated RBCs (Gregory and Eaves, 1978; Metcalf, 1993; Giarratana et al., 2005). As dyserythropoiesis is implicated in the pathogenesis of severe malaria, such *ex-vivo* models of erythropoiesis also hold potential for advancing our understanding of host-pathogen interactions unique to the hematopoietic niche.

## *Ex-vivo* Erythropoiesis of Primary Cells

Early *ex-vivo* liquid culture systems using primary CD34^+^ cells demonstrated a high proliferative capacity in culture in the presence of cytokines, but efficient terminal differentiation and enucleation proved challenging (Fibach et al., 1989; Wada et al., 1990; Sui et al., 1996; Panzenbock et al., 1998; Freyssinier et al., 1999; von Lindern et al., 1999). A significant advance came with the development of an approach enabling the large-scale production and detailed characterization of RBCs generated from primary human CD34^+^ HSPCs using a three-phase protocol (Giarratana et al., 2005). This involved mimicking the microenvironment of the hematopoietic niche through addition of cytokines such as SCF and EPO, and co-culture on a murine stromal layer to promote enucleation. Importantly, the cultured progenitors were observed to differentiate normally down the erythroid lineage as measured by morphology and cell surface markers, and the terminally differentiated, cultured RBCs (cRBCs) had similar enzyme content and functional hemoglobin as natural adult RBCs.

In subsequent work, a modified approach that enhanced the feasibility of large-scale production of RBCs by removing the need for co-culture on a stromal layer was reported (Giarratana et al., 2011). This advance was achieved using human plasma, which was hypothesized to provide some of the necessary extracellular signals to promote survival, terminal differentiation and enucleation. While the resulting RBCs were more similar to reticulocytes than mature erythrocytes based on cell volume, membrane deformability, and surface protein expression, they matured to erythrocytes when transferred back to NOD/SCID mice or a human donor. This study provided strong experimental evidence for the quality and functionality of this *ex-vivo* approach for erythroid differentiation. Further validation of this *ex-vivo* erythropoiesis model has come from its application to the study of *P. falciparum*, in which genetic manipulation of erythroid progenitors followed by terminal differentiation enabled discovery and interrogation of novel host factors in enucleated cRBCs (Egan et al., 2015; Shakya et al., 2021).

Other potential sources of primary cells to model human erythropoiesis include human cord blood, embryonic stem cells (hESC), and induced pluripotent stem cells (iPSCs), but none have the demonstrated utility of primary adult CD34^+^ HSPCs. Human cord blood is a readily available source of CD34^+^ HSPCs, but these are fetal cells that predominantly generate HbF rather than adult hemoglobin (Anstee et al., 2012). hESC have high proliferative potential, but limited ability to enucleate and express adult hemoglobin. iPSC approaches are hampered by limited proliferative capacity, inefficient terminal differentiation, and presence of fetal or embryonic rather than adult hemoglobin (Dias et al., 2011; Trakarnsanga et al., 2014). More recent research suggests that combining insights from human genetic variation with advances in genome editing can help optimize proliferation and erythroid differentiation of hESC and iPSC (Giani et al., 2016).

## Erythroid Cell Lines

Initial *in vitro* models of erythropoiesis made use of erythroleukemic cancer cell lines (Friend et al., 1971; Tsiftsoglou et al., 2009). While valuable for their reproducible growth and ease of genetic manipulation, many cancerous cell lines do not recapitulate important aspects of healthy, adult erythropoiesis, in terms of responsiveness to EPO-signaling, hemoglobin expression, and efficiency of terminal differentiation and enucleation (Tsiftsoglou et al., 2009). One promising cell line is the JK-1 erythroleukemia cell line, which can be induced to differentiate using bromodomain inhibitors (Kanjee et al., 2017). JK-1-derived erythroblasts have been shown by morphological analyses to resemble established erythroid progenitor populations, and quantitative plasma membrane proteomics of JK-1 cells predominantly at the polychromatic erythroblast stage revealed that their proteome resembled that of polychromatic erythroblasts derived from primary human HSPCs, with ~68% of proteins within a 2-fold equivalent range of relative abundance. While JK-1 cells do not enucleate, they can support invasion by *P. falciparum*, and have some demonstrated utility for the genetic analysis of erythrocyte receptors for malaria (Kanjee et al., 2017).

## Models of Human Erythropoiesis From Immortalized Cell Lines

Immortalized erythroid progenitor cell lines derived from CD34^+^ HSPCs offer alternative models of healthy erythropoiesis that may assuage some of the technical challenges and expense associated with primary cells (**Table 1**). Cell lines can be cloned, genetically manipulated, and grown to high numbers in culture, ideally yielding an unlimited source of reproducible material for erythropoiesis-focused applications and molecular investigations. In recent years, several immortalized erythroid cell lines have been reported, with continued improvements toward their ability to recapitulate healthy erythropoiesis, yet limitations to their applicability still persist.

**Table 1:** Summary of models for *ex-vivo* erythropoiesis.

Model		Strengths	Limitations	Prior use in *Plasmodium*	References
Erythro-leukemic cell line	JK-1	Unlimited proliferation Ease of genetic engineering Heterogeneity can be reduced epigenetically	Poor enucleation Differentiation only to polychromatic erythroblast stage	Used as model for peripheral blood RBCs to study host determinants of invasion	(Okuno et al., 1990; Kanjee et al., 2017)
Primary HSPC	CD34+ from bone marrow or peripheral blood	Efficient *ex-vivo* erythropoiesis in liquid culture mirrors *in vivo* development All erythroblast stages can be generated Efficient enucleation	Limited proliferative capacity (~10,000-fold) Optimal terminal differentiation requires co-culture on stromal layer High heterogeneity Genetic engineering is technically challenging	Used as model for peripheral blood RBCs to study host determinants of invasion Used to define erythroblast populations susceptible to *Pf* infection Bulk transcriptomic analysis of *Pf*-infected erythroblast cultures Supports gametocyte formation	(Tamez et al., 2009; Bei et al., 2010; Giarratana et al., 2011; Tamez et al., 2011; Joice et al., 2014; Egan et al., 2015; Neveu et al., 2020; Shakya et al., 2021)
	CB	Readily available Efficient *ex-vivo* erythropoiesis in liquid culture All erythroblast stages can be generated Efficient enucleation	Limited proliferation Optimal terminal differentiation requires co-culture on stromal layer Expresses fetal hemoglobin High heterogeneity Genetic engineering is technically challenging	Culture of *P. vivax* Effects of co-culture with *P. falciparum* on expression of globin genes Effects of Hz on erythropoiesis	(Casals-Pascual et al., 2006; Panichakul et al., 2007; Anstee et al., 2012; Pathak et al., 2018)
	hESC, iPSC	High proliferative capacity HbF expression may be useful therapeutically (e.g. sickle cell) Feasible for targeted genomic modification	Poor enucleation Optimal terminal differentiation requires co-culture on stromal layer Expresses predominantly fetal hemoglobin (HbF), although switching to adult Hb observed *in vivo* in NOD/SCID mice	N/A	(Hanna et al., 2007; Dias et al., 2011; Trakarnsanga et al., 2014; Giani et al., 2016)
Immortalized cell line	HUDEP-2	High proliferative capacity Low heterogeneity Ease of genetic engineering Adult hemoglobin	Poor enucleation and loss of viability at the orthochromatic erythroblast stage	N/A	(Kurita et al., 2013)
	BEL-A	High proliferative capacity Low heterogeneity Ease of genetic engineering	Modest enucleation and loss of viability at the orthochromatic erythroblast stage	Used as model for peripheral blood RBCs to study host determinants of invasion	(Trakarnsanga et al., 2017; Satchwell et al., 2019; Daniels et al., 2020)

The HUDEP-2 erythroid progenitor cell line was generated from human umbilical cord blood CD34+ HSPCs (Kurita et al., 2013). These cells were immortalized using the doxycycline-inducible HPV16-E6/E7 construct after initial growth in a non-erythroid specific medium. Incubation in defined media including SCF, EPO, dexamethasone, and doxycycline enabled continued proliferation, and transitioning to differentiation media with EPO alone induced differentiation down the erythroid lineage. This cell line has been characterized to some degree in terms of transcription factor and cell surface protein expression, and expresses functional ß-globin. Though HUDEP-2 have the potential to enucleate, the rate of enucleation is low (5-20% depending on the media used) and significant cell loss is seen as cells reach the orthochromatic erythroblast stage, suggesting terminal differentiation is not fully functional in these cells (Kurita et al., 2013; Daniels et al., 2020).

In 2017, the first erythroid cell line immortalized from adult bone marrow CD34^+^ HSPCs was reported, termed BEL-A (Trakarnsanga et al., 2017). BEL-A were initially maintained in an optimized erythroid culture medium, and then immortalized using the same doxycycline-inducible HPV16-E6/E7 construct as was used for HUDEP-2. The BEL-A cells have been shown to proliferate at similar rates as HUDEP-2, but they differentiate more quickly and have a higher enucleation rate, up to 40% (Daniels et al., 2020). However this enucleation rate is still modest relative to primary cell cultures, and BEL-A suffers a viability loss similar to HUDEP-2 after ~ 8 days of differentiation, suggesting a defect in the transition from orthochromatic erythroblasts to reticulocytes. Functionally, BEL-A have been used to study erythrocyte proteins required for *P. falciparum* invasion, and were used to further validate basigin as an essential receptor for malaria (Satchwell et al., 2019).

Recently, Daniels et al. showed that the immortalization approach used to make BEL-A could be adapted to erythroblasts from various sources, including human bone marrow, peripheral blood, and cord blood (Daniels et al., 2020). Through characterization of the proteome of immortalized cells derived from different sources compared to primary cells, a molecular signature for immortalization began to emerge, involving cell cycle regulation. Importantly, the progenitors immortalized using this approach were shown to recapitulate primary cells in terms of differentiation potential and adult hemoglobin expression levels (Daniels et al., 2020).

## Application of *in vitro* Models to Study Malaria in the Hematopoietic Niche

*Ex vivo* erythropoiesis of primary cells presents an opportunity to study host responses and parasite development within in the hematopoietic niche. Much remains to be understood about the host response to exposure with *P. falciparum*, and how it might lead to disordered erythropoiesis. Recent work in the field has begun to elucidate the effects of *P. falciparum* and the heme degradation product hemozoin (Hz) on gross, phenotypic measurements of erythroid development, including proliferation, cell cycle staging, and expression of key erythroid-related genes.

Hz is found in large quantities in malaria-infected bone marrow, both in the extracellular environment and within phagocytic monocytes (Casals-Pascual et al., 2006; Aguilar et al., 2014b; Joice et al., 2014). Several studies have used *ex vivo* culture of erythroid precursors to study the effect of Hz on erythropoiesis. The addition of Hz, or conditioned media from monocytes fed with Hz, to primary cell cultures of BFU-E/CFU-E reduced colony formation (Giribaldi et al., 2004; Skorokhod et al., 2010). Similarly, periodic addition of Hz to primary CD34+ cells during differentiation down the erythroid lineage reduced cell growth (Casals-Pascual et al., 2006; Skorokhod et al., 2010). Co-cultivation with Hz also resulted in cell cycle defects in primary cells and changes to protein expression of cell cycle markers p53, p21, and Cyclin A in K562 cells (Skorokhod et al., 2010). This result provides one possible explanation for the observation of cell cycle defects in bone marrow aspirates from malaria patients. In a study using microarray-generated gene expression profiles, incubation of primary erythroblasts with Hz was shown to increase expression of some stress response genes, including those that mediate apoptosis (Lamikanra et al., 2015). Together, the current evidence supports a role for Hz in disordered erythropoiesis in the bone marrow.

*Ex vivo* erythropoiesis has also been used to investigate direct interactions between *P. falciparum* and developing erythroblasts. A study on the use of *in vitro*-produced reticulocytes for culture of *P. vivax* observed ring-stage parasites and gametocytes inside nucleated erythroid precursors by Giemsa staining (Panichakul et al., 2007). This observation was also confirmed for *P. falciparum* asexual (Tamez et al., 2009) and sexual stage parasites (Joice et al., 2014; Neveu et al., 2020). These studies further demonstrated that invasion and development of the parasite were dependent on erythroblast stage. Neveu *et al*. leveraged a GFP-expressing parasite line and host markers for erythroid development to show by flow cytometry that erythroid cells as early as the basophilic erythroblast are susceptible to parasite invasion. The authors discovered two indicators of dyserythropoiesis that are specific to infected cells, reduced enucleation rate and generation of reactive oxygen species. Intriguingly, this study also demonstrated the presence of infected, nucleated erythroid precursors in bone marrow aspirates of malaria patients by immunofluorescence.

Little is known about the gene expression response to *P. falciparum* in infected erythroblasts. Through microarray profiling, Tamez et al. provided some evidence that 24-hr co-culture with *P. falciparum* was associated with transcriptional upregulation of a number of host genes in late-stage erythroblasts. This includes changes to genes encoding transcription factors related to erythroid development (JUN, MYC, SOX6) and mediators of the cellular stress response (HMOX1, DNAJB1, HSPA1A, HSP90AB1, STIP1) (Tamez et al., 2011). However, the conclusions from this study were limited, in part because the methodology did not distinguish between infected versus bystander cells, and the erythroblasts were heterogeneous in terms of developmental stage.

## Complementary Approaches *in vivo*

Although there are advantages to the reductionist approach of cell culture models, erythroid cells in the bone marrow respond to complex combinations of local and systemic signals that cannot be easily or fully reconstituted *in vitro*. Studies on anemia of inflammation show that erythropoiesis is modulated by activity of the immune system, including by production of cytokines that act on erythroid precursors and immune-regulated changes in iron availability (Hom et al., 2015). While chemical signals can be added exogenously in *ex-vivo* cultures, reproducing inter-organ signaling is not straightforward. For example, IL-6, a proinflammatory cytokine that is elevated in malaria infection, regulates expression of hepcidin in the liver, which controls the flow of iron to developing RBCs in the bone marrow (Nemeth et al., 2004). Primary HSPC cultures are also limited in their utility for modeling spatiotemporal aspects of infection and may have issues of cost and reproducibility due to dependence on donors, and inherent donor-to-donor variability.

Animal models, especially small rodents, have long been an invaluable tool for studies of the hematopoietic niche that are not easily pursued in humans or with cell culture experiments (Doulatov et al., 2012). *In vivo* models preserve the spatial organization, signaling, and physical properties of the diverse cell types that define the bone marrow niche. No single murine model recapitulates all aspects of human malaria; however, the numerous combinations of host and parasite strains enable interrogation of a wide range of mechanistic questions about malarial pathogenesis. Excellent reviews on leveraging rodent malaria as an experimental system are available elsewhere (Lamikanra et al., 2007; De Niz and Heussler, 2018).

Murine models have extended insights from post-mortem studies and *in vitro* models of host-parasite interactions in the hematopoietic niche. One major avenue of investigation is in how, when, and at what stage parasites arrive in the bone marrow and exit into circulation. Intravital imaging during murine infection with a gametocyte-forming, fluorescent strain of *Plasmodium berghei* found gametocytes specifically accumulated in the extravascular space, arriving early in infection (De Niz et al., 2018). The same work demonstrated that gametocytes were enriched in RBC precursors in the bone marrow after 24 hours of infection and that mature gametocytes could translocate across the endothelial barrier from the bone marrow into circulation (De Niz et al., 2018). Infection with *P. berghei* has also revealed that parasites invade nucleated erythroblasts *in vivo* as well as enucleated, early reticulocytes (Lee et al., 2018). Single cell RNA-seq (scRNA-seq) of bone marrow HSPCs from *P. berghei-*infected mice revealed a shift in lineage commitment toward the myeloid and basophil lineages at the expense of erythroid and megakaryocyte production, revealing a potential mechanism of impaired red cell production during infection (Haltalli et al., 2020). In another study of *P. berghei* infection in the hematopoietic niche, infected cells from the spleen, bone marrow, and liver were analyzed by CITE-seq (Cellular Indexing of Transcriptomes and Epitopes by Sequencing) using antibodies against markers of erythroid maturation (Hentzschel et al., 2022). The authors found differences in parasite metabolism and likelihood of gametocyte commitment based on the maturation state of the host cell. Animal models are also key for investigating early infection events in the exoerythrocytic stage, which have received less attention in recent studies on the bone marrow. The finding that the hematopoietic niche responds to natural infection in the liver stage of *P. berghei* infection, including by increasing proliferation of HSC and loss of myeloid-committed progenitors, warrants further investigation of hematopoietic changes during the liver stage (Vainieri et al., 2016).

Mouse malaria has also provided insight into interactions between developing erythroid cells and the immune system. Infection with GFP-expressing *Plasmodium yoelii* yielded parasitized, nucleated erythroblasts in the bone marrow, again suggesting that infection of erythroid precursors is a generalized aspect of pathogenesis across *Plasmodium* species. Isolation of parasitized erythroblasts from infected mice showed that infected erythroid precursors express MHC class I at the cell surface and are capable of activating CD8^+^ T cells. Several studies investigated dyserythropoiesis in the context of severe malaria anemia (SMA) using rodent malaria; however, the results are difficult to interpret due to differences in erythropoiesis and the manifestation of severe malaria in mice and humans (Lamikanra et al., 2007). Increased use of non-human primates to model malaria is a promising solution (Craig et al., 2012). New World monkeys belonging to the genus *Aotus* and *Saimiri* are infectable with *P. vivax* and histological studies of post-mortem tissue show parasites accumulated in the bone marrow (Obaldia et al., 2018). Vaccinated, semi-immune *Aotus* has also been suggested as model of SMA with features that are analogous to infection in children living in malaria-endemic areas (Egan et al., 2002). In contrast to mice, infection at low parasitemia produced severe malaria symptoms including bone marrow suppression (Egan et al., 2002).

## Future Perspectives

Existing models for *ex vivo* erythropoiesis, in combination with novel transcriptomic and proteomic strategies, have potential to provide a detailed picture of the host response to *P. falciparum* in erythroid progenitor cells. To date, host transcriptional responses in co-culture of *P. falciparum* and erythroblasts have only been studied in bulk. Improved resolution is needed to remove confounding factors from bulk readouts in an environment that contains a mix of cell types as well as uninfected and infected cells. Advances in surface phenotyping of developing erythroblasts by flow cytometry could be used to sort cell populations before sequencing (Hu et al., 2013; Li et al., 2014; Yan et al., 2018; Yan et al., 2021). Single cell RNA-seq (scRNA-seq) enables transcriptomic analysis of heterogeneous cell populations on the level of individual cells, and has yet to be applied to *Plasmodium*-infected erythroblasts in a human system. Single cell techniques at the protein-level could also be used to overcome artifacts from bulk analysis. Mass cytometry is an increasingly utilized way of monitoring expression of proteins that reflect complex cellular phenotypes and functions. Mass cytometry, also called Cytometry by Time of Flight (CyTOF), is a variation of flow cytometry in which elemental (heavy metal) isotopes are conjugated to antibodies in the place of fluorophores to allow dozens of markers to be analyzed simultaneously (Hartmann and Bendall, 2020). CyTOF has been successfully applied in many studies of hematopoiesis and to a limited extent, erythropoiesis (Thomson-Luque et al., 2018; Palii et al., 2019). This technology could be applied to study dyserythropoiesis in malaria infection, especially with the addition of a parasite marker to distinguish infected cells. The application of single cell technology to existing models would add to our understanding of host responses to parasite infection at different stages of erythropoiesis and generate testable hypotheses about mechanisms of dyserythropoiesis related to direct and indirect interactions between host and parasite.

The bone marrow is a challenging tissue to model *in vitro* because of its complex architecture, heterogeneous microenvironments, and diverse cell types. Current *in vitro* studies of *Plasmodium* in the hematopoietic niche are primarily limited to interactions with a single lineage, usually in 2D culture. To improve *in vitro* models of complex tissues and reduce reliance on animal research, there is growing interest in microfluidic organ-on-a-chip (OOAC) devices that are capable of mimicking physical cues, such as shear flow and mechanical stress, that regulate cell growth and differentiation (Wu et al., 2020; Li et al., 2021). In the study of malaria pathogenesis, OOACs have primarily been used to study how physical parameters of infected RBCs influence adhesion and accumulation in the microvasculature (Baddal and Marrazzo, 2021). Unlike capillary models, bone marrow-on-a-chip (BMoC) requires maintaining multiple cell types and a delicate balance of cytokines to coordinate self-renewal, proliferation, and lineage commitment of HSCs.

Recent methods for engineering BMoC have made promising advances for *in vitro* modeling of the hematopoietic niche. The first successful BMoC involved *in vivo* implantation of a device in murine bone marrow where new bone and blood-filled marrow would form that could then be removed and transferred to a microfluidic device for culture (Torisawa et al., 2014). The authors showed that the engineered bone marrow in culture contained differentiated blood cell lineages in similar proportions to *in vivo* bone marrow over 7 days of culture and that HSCs retained their self-renewal and lineage potential *in vitro* (Torisawa et al., 2014). Such a system would be useful for visualizing cell-cell interactions in co-culture with *Plasmodium* parasites enables the study of immune cell activity in the bone marrow in response to parasite infection. There has also been success with a human BMoC perfused device containing one chamber for 3D co-culture of CD34^+^ cells and bone marrow stromal cells and a second chamber for culture of endothelial cells (Chou et al., 2020). The authors report proliferation of hematopoietic cells over 28 days of culture and differentiation down the erythroid and myeloid lineages. Interestingly, mature neutrophils and cells of the myeloid lineage intravasated into the chamber containing endothelial cells, mimicking exit of cells into the peripheral blood (Chou et al., 2020). This model would be especially useful for the study of factors involved in entry and exit of gametocytes in the bone marrow.

Although advancements in *ex vivo* culture of primary HSPCs hold promise, it is still important to consider host-parasite interactions in the context of human infection. An ideal animal model would allow study of human malaria species, particularly *P. falciparum* for investigation of chronic anemia of malaria. So-called “humanized” mice are a potential solution to the non-analogous aspects of murine erythropoiesis and malaria infection. Humanized mice have been successfully used to study accumulation of *P. falciparum* in deep tissue and test chemotherapy against sequestered gametocytes (Duffier et al., 2016). Such models have yet to be used in the study of dyserythropoiesis during malaria infection. The application of humanized mice would answer questions about the *in vivo* relevance of infection of erythroid progenitors and pave the way for studies of host responses related to the erythroid lineage.

## Conclusions

A variety of approaches have been developed to model human erythropoiesis, enabling the study of *Plasmodium* host-parasite interactions in the hematopoietic niche. *Ex vivo* erythropoiesis of human primary HSPCs has provided important insights into gametocyte commitment and characterization of *Plasmodium* infection of a non-canonical host cell type, and holds potential as a powerful tool for dissecting host responses and mechanisms of impaired erythropoiesis during malaria infection. In combination with *in vivo* models, *ex vivo* erythropoiesis can be leveraged to provide insight into key aspects of malaria pathogenesis and transmission. As host-parasite interactions in the hematopoietic niche come into focus, so will opportunities for therapeutic interventions for dyserythropoiesis and strategies to eliminate transmission of malaria.

## Author Contributions

TF and EE designed and conceptualized the manuscript, carried out the literature search, generated the first draft of the manuscript, and reviewed and approved the final manuscript.

## Funding

This work was supported by the National Institutes of Health under Award Numbers T32GM007276 (TF) and 1DP2HL13718601 (EE), and by a Burroughs Wellcome Fund PATH Award, under Award Number 1021370 (EE).

## Conflict of Interest

The authors declare that the research was conducted in the absence of any commercial or financial relationships that could be construed as a potential conflict of interest.

## Publisher’s Note

All claims expressed in this article are solely those of the authors and do not necessarily represent those of their affiliated organizations, or those of the publisher, the editors and the reviewers. Any product that may be evaluated in this article, or claim that may be made by its manufacturer, is not guaranteed or endorsed by the publisher.
